# Comparing Bioenergy Production Sites in the Southeastern US Regarding Ecosystem Service Supply and Demand

**DOI:** 10.1371/journal.pone.0116336

**Published:** 2015-03-13

**Authors:** Markus A. Meyer, Tanzila Chand, Joerg A. Priess

**Affiliations:** Department Computational Landscape Ecology, UFZ – Helmholtz Centre for Environmental Research, Leipzig, Germany; Chinese Academy of Forestry, CHINA

## Abstract

Biomass for bioenergy is debated for its potential synergies or tradeoffs with other provisioning and regulating ecosystem services (ESS). This biomass may originate from different production systems and may be purposefully grown or obtained from residues. Increased concerns globally about the sustainable production of biomass for bioenergy has resulted in numerous certification schemes focusing on best management practices, mostly operating at the plot/field scale. In this study, we compare the ESS of two watersheds in the southeastern US. We show the ESS tradeoffs and synergies of plantation forestry, i.e., pine poles, and agricultural production, i.e., wheat straw and corn stover, with the counterfactual natural or semi-natural forest in both watersheds. The plantation forestry showed less distinct tradeoffs than did corn and wheat production, i.e., for carbon storage, P and sediment retention, groundwater recharge, and biodiversity. Using indicators of landscape composition and configuration, we showed that landscape planning can affect the overall ESS supply and can partly determine if locally set environmental thresholds are being met. Indicators on landscape composition, configuration and naturalness explained more than 30% of the variation in ESS supply. Landscape elements such as largely connected forest patches or more complex agricultural patches, e.g., mosaics with shrub and grassland patches, may enhance ESS supply in both of the bioenergy production systems. If tradeoffs between biomass production and other ESS are not addressed by landscape planning, it may be reasonable to include rules in certification schemes that require, e.g., the connectivity of natural or semi-natural forest patches in plantation forestry or semi-natural landscape elements in agricultural production systems. Integrating indicators on landscape configuration and composition into certification schemes is particularly relevant considering that certification schemes are governance tools used to ensure comparable sustainability standards for biomass produced in countries with variable or absent legal frameworks for landscape planning.

## Introduction

Research in the context of bioenergy and ecosystem services (ESS), the perceived human benefits from ecological systems [1], often focuses on largely debated 1^st^ generation liquid biofuel feedstocks such as maize in the US, sugarcane or soybeans in Brazil, or rapeseed in Europe [2]. Some papers address scenarios with a shift to 2^nd^ generation liquid biofuel feedstocks, such as grasses or other perennial bioenergy feedstocks [3,4]. Research in this area only partly reflects the fact that only 3% of the global bioenergy supply was obtained from dedicated energy crops in 2008. More than 80% of the global bioenergy supply originates from forest biomass [5]. With respect to modern solid bioenergy carriers, wood pellets have experienced an increased global trade volume, accounting for 120 PJ (∼660 Mt) of the total global solid bioenergy carrier trade of 300 PJ (∼1640 Mt) as of 2010 [6]. For trade between EU and non-EU countries in 2010, the wood pellet trade volume of 45 PJ (∼250 Mt) is comparable to those of biodiesel and bioethanol [7].

Increasing forest biomass use and trade may also affect the supply of other ESS, e.g., carbon storage or groundwater recharge [8,9], or create environmental impacts exceeding the capacity of regulating ESS; e.g., increasing biomass may affect sediment retention due to increased plantation forestry [10]. The expansion of bioenergy production is limited by and competing with the demand for land for other bio-based commodities (food, feed and fiber) [11]. In that respect, a current draft of new sustainability requirements of the EU Renewable Energy Directive (RED) emphasizes the consideration and quantification of tradeoffs of feedstock production for liquid, gaseous and solid bioenergy and other ESS, such as carbon storage or sediment retention [12]. Further research in this context may examine the ability to avoid negative impacts, such as the effects on erosion, carbon storage or biodiversity [2,7,13].

Existing studies on ESS supply typically model a single case study area with a mostly heterogeneous or contrasting land use/land cover composition and partly model synergies and tradeoffs in ESS supply, e.g., [14,15]. Rather homogenous and more intensively managed land use/land cover systems for ESS supply, e.g., systems specialized in forest plantations or agriculture, may require more significant tradeoffs regarding other ESS compared to heterogeneous production systems for biomass. For example, they are more likely to exceed critical environmental thresholds such as erosion control, water purification or recreation due to underrepresented natural or semi-natural vegetation [16,17]. Increased landscape heterogeneity helps to ensure a balanced supply of biodiversity and regulates ESS, such as the higher nutrient retention efficiency of riparian buffer zones in agricultural landscapes [18]. Larger quantities of bioenergy feedstocks may generate economies of scale for processing and logistics [19], contributing to more homogenous landscapes. Considering that different biomass provision options may become even more important in the near future, we analyze ESS supply both in forest plantations and agricultural systems, which may be used interchangeably. For example, agricultural residues, such as cereal straw or corn stover, currently amount to 4% of the global bioenergy supply [5]. In the US, cereal straw and corn stover comprise 97% of the estimated available agricultural residues in the US (2011) [20]. Using residues contributes to reducing or completely avoiding the food versus fuel conflict compared with dedicated energy crops [21]. Direct land use change (LUC) is when biomass production replaces other crops, forests or natural grasslands. Indirect land use change (iLUC) is the clearing of land not specifically for biomass but to meet the demands for other commodities, such as food and fiber, and may occur not only nearby but also in different parts of the region or even different parts of the world [22].

A wide range of factors may influence ESS supply, such as environmental conditions, including the topography, soil characteristics and climate. In contrast, land management may affect ESS supply [23–25]. In the context of bioenergy production, certification schemes are used as a governance tool to ensure sustainable production. They focus on indicators and prescribed management practices mostly applicable at the plot scale [26,27]. However, certification schemes rarely require indicators at the regional or landscape scale in the context of both bioenergy [12] and agricultural products [28] and payment schemes for ESS [29]. At the landscape/regional scale, i.e., the typical scale of landscape planning, the influence of landscape composition and configuration has been argued [30,31] and exemplarily demonstrated for single ESS, i.e., soil protection and retention [32] and biodiversity [33].

In this paper, we first assess ESS supply in subtropical watersheds mostly used for (i) forest plantations, *Pinus* spp., and (ii) agricultural production as bioenergy sourcing regions in the southeastern US. Following [17,34], we expect that the tradeoffs between forest plantations and natural or semi-natural forest as a counterfactual are smaller than between corn and wheat production and natural or semi-natural forest. The remnants of the existing forests reflect the potential natural vegetation in both watersheds [35]. Second, we hypothesize that not only environmental or management factors at the plot scale but also landscape composition and configuration and naturalness assessed at the landscape scale influence ESS supply and biodiversity. Third, we assume that these landscape factors play a role in whether socially accepted environmental thresholds, e.g., water quality, are met within the two contrasting case studies. For example, the connectivity or dominance of patches of natural land cover, which may serve a buffering function, strengthens nutrient or sediment retention.

## Materials and Methods

### Study sites

**Fig 1:**
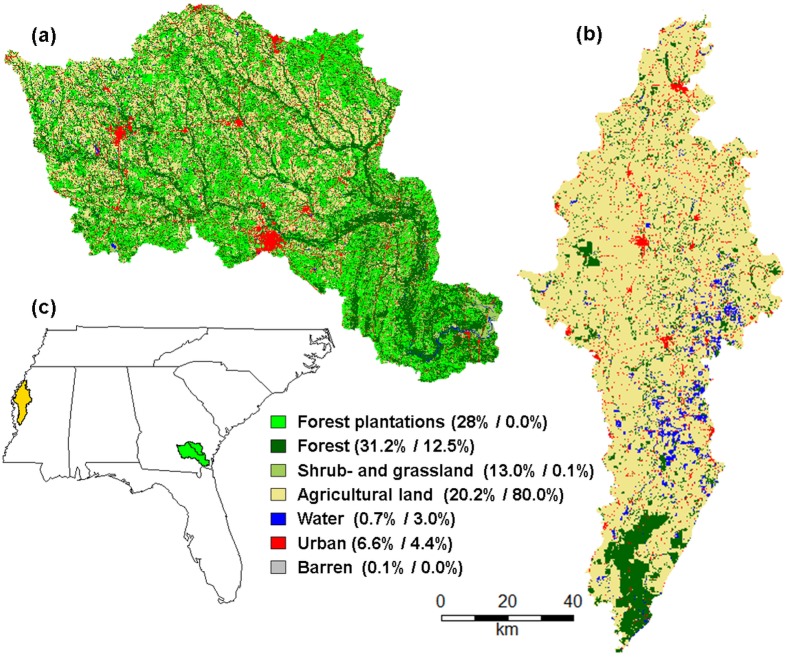
Land use/land cover in the Satilla (a) and Big Sunflower (b) watersheds and their location in the southeastern US (c) [61,62].

The decline in pulpwood demand in the pulp and paper industry released capacities of existing pine plantations for wood pellets in the southeastern US [36]. The 2008/09 recession and decline of the housing market released round wood from the timber market for solid bioenergy production [6]. A large share of up to 80 PJ (∼440 Mt) of the produced pellets is expected to be exported to the EU by 2020 [6,7]. The Big Satilla and Little Satilla watersheds, addressed as the Satilla watershed throughout the paper, are representative examples of such pine plantation production systems in a humid subtropical climate. The Satilla watershed includes an area of 8,760 km² (hereof: 28% forest plantations in 2006, see Fig. 1) and is located in southeast Georgia, US.

For agricultural residues as an alternative feedstock option, the Mississippi Delta in humid subtropical western Mississippi, US is one of the major agricultural production areas in the subtropical southeastern US due to its alluvial fertile soils. The commodities include corn and wheat [37], with the area producing 68% of the winter wheat and 79% of the corn in Mississippi in 2013 [38]. A common practice for residues is to burn them completely onsite. Alternatively, a certain share of residues may be used for bioenergy without negatively affecting the nutrient and carbon balances [39]. The Big Sunflower watershed covers 8,170 km² (hereof: 80% agricultural land (four percentage points corn and winter wheat production) in 2006), which represents most of the Mississippi Delta. The Big Sunflower River is a major river in the Yazoo River basin; the latter is a tributary of the Mississippi river.

We model ESS supply for 2006, for which land cover information differentiating between natural and semi-natural forest and plantation forestry is available. Climatically, the precipitation in 2006 in the Big Sunflower watershed (1276.9 mm, SD: 31.0 mm) was 7% lower than the normal climate conditions for the period from 1981 to 2010 [40]; the precipitation in the Satilla watershed (950.7 mm, SD: 63. 9 mm) was 22% lower than the normal climate conditions. The minimum and maximum average temperatures in 2006 in the Big Sunflower watershed (T_min_: 11.9°C, SD: 0.3°C, T_max_: 24.2°C, SD: 0.5°C) and in the Satilla watershed (T_min_: 11.9°C, SD: 0.3°C, T_max_: 26.4°C, SD: 0.2°C) deviated less than 1 degree Celsius from the normal climate conditions for the period from 1981 to 2010 [40].

### Ecosystem services

ESS are classified as provisioning, regulating and cultural ecosystem services [41]. We identified the following potentially affected provisioning and regulating ESS based on the existing literature on environmental impacts and ESS for bioenergy [13,27,42–44] and in general [14,18,24,45,46]: carbon storage, nutrient retention, sediment retention, and groundwater recharge. In addition, we assess the impact on biodiversity, which may be a recreational ESS in itself but largely supports the supply of other ESS [47,48]. We focus on phosphorous (P) retention because (i) the nutrient retention efficiency is higher than that for nitrogen, (ii) agricultural sources are responsible for approximately 80% of the P input in the Gulf of Mexico, and (iii) P has been underestimated in its contribution to the eutrophication of the Gulf of Mexico for the Big Sunflower watershed region [46].

Sustainable production of bioenergy will only be possible if a feedstock is available without major negative direct and indirect impacts on ESS and biodiversity. In contrast to the plantation forestry system with alternative uses of timber, e.g., as construction wood, corn stover and wheat straw have no competing use but are burnt onsite in the Mississippi Delta and are therefore unlikely to cause iLUC risks. Therefore, residue use for bioenergy may save GHG emissions, which are not covered in the ESS assessment. We calculate the amount of sustainably available agricultural residues, i.e., from corn and winter wheat, based on 2006 production data [38], on ranges of sustainable harvest residue removal rates [39,49,50], and average CO_2_ and CH_4_ emission factors for onsite burning practices for agricultural residues in the US [51].

#### Carbon storage

The amount of carbon stored was modeled with InVEST (Integrated Valuation of Environmental Services and Tradeoffs) [52,53] by refining standard assumptions for aboveground [54–57], belowground [14,55,56,58], soil [54,57–60] and dead organic carbon [14,57,60] for the land use/land cover data for 2006 [61,62].

#### Phosphorous retention

The amount of retained P was modeled with InVEST. The model estimates P export and retention to surface water bodies based on the land use/land cover specific P input and retention capacity as well as the water yield. The major spatial inputs are the land use/land cover data for 2006 [61,62], a digital elevation model (DEM) [63], annual precipitation data for 2006 [40], and the long-term annual average reference evapotranspiration [64] as well as the depth to any root restrictive layer and the available water holding capacity (AWC) from the Soil Survey Geographic (SSURGO) database [65]. We modified the InVEST default assumptions for evapotranspiration coefficients [66,67], rooting depth [68–72], P export rates and P retention efficiencies [73–75]. We validated the modeled P export against the corresponding phosphorous concentration measurements of the stations *Little Satilla near Offerman (USGS 02227500)*, *Satilla River at Atkinson (USGS 2228000)* and *Big Sunflower River at Sunflower (USGS 07288500)*.

#### Sediment retention

The amount of retained sediment was modeled with InVEST. The model estimates the sediment retention and export based on the modeled soil loss from the universal soil loss equation (USLE) [76,77], i.e., sheet erosion, and the land use/land cover specific sediment removal efficiencies. The same land use/land cover and DEM datasets used for modeling P retention were used. We obtained the k factor (soil erodibility) from the SSURGO database [65]. We calculated the r factor (rainfall erosivity) based on the following formula [78]:

$$R=\left(210+89*log_{10}I_{30}\right)*I_{30}$$

where *I*_*30*_ is the maximum rainfall intensity in 30 minutes obtained from [79]. We refined the required cover and management factor *C* [77,80–85] and the support practice factor *P* [80,82,84,86]. We validated the model outcome against the suspended sediment concentration for the same stations as for P export.

#### Groundwater recharge

The net infiltration was modeled with the soil-water balance model (SWB) from the USGS [87,88]. We simulated the groundwater recharge on a daily basis with the Thornthwaite-Mater evapotranspiration calculation method. We used the same land use/land cover, DEM and AWC datasets as when modeling P retention. The hydrologic soil groups were obtained from the SSURGO database [65]. The daily average temperature and precipitation data were obtained for the *Waycross 4 NE (USC00099186)* (Satilla watershed) and *Cleveland (USC00221738)* (Big Sunflower watershed) stations [89]. We validated the results with a spatially explicit study on groundwater recharge, a modeled average for the period from 1951 to 1980, for the conterminous US with a resolution of one km [90] and with two other studies [91,92] with more recent indicative ranges of groundwater recharge for larger regions including the targeted watersheds.

#### Biodiversity

A spatially explicit dataset for biodiversity was used to model terrestrial vertebrate species richness resulting from the GAP Analysis program from the USGS for Georgia [93] and Mississippi [94].

### Tradeoff analysis

To identify tradeoffs in ESS supply, we distinguished the following major land use/land cover classes, as adapted from [95–97]:

1. Natural or semi-natural forest (counterfactual)
2. Plantation forestry (only the Satilla watershed)
3. Corn and winter wheat (only the Big Sunflower watershed)
4. Agricultural land (other)

We calculated the arithmetic mean ESS supply for these major land use/land cover classes and normalized them to the maximum value in each ESS category for each watershed. We conducted this analysis to assess the differences between targeted land use/land cover classes. In addition, the paired Pearson correlation coefficients between the ESS, selected from the list of methods in Mouchet et al. [98], were calculated for the entire watershed with the statistical software package R [99] to assess general ESS and biodiversity trade-offs in current production systems specialized in plantation forestry and agriculture respectively.

Because we considered two significantly different land use systems, it was reasonable to have a counterfactual, which served as a baseline to compare several alternatives of natural or semi-natural forest. This approach is recommended to test the suitability of bioenergy feedstock production options in the local hydrological context [100] or for biodiversity [101].

### Indicators at the plot and landscape scale that potentially explain variation in ecosystem service supply

In this study, we tested indicators of landscape composition, configuration and naturalness for their influence on ESS supply in both watersheds (see Table 1). Therefore, we calculated the landscape composition and configuration indicators in a moving window approach for a buffer of 300 m, which was ten times the minimum pixel size [14]. At the plot scale, potential explanatory variables of topography and soil properties were used to set the explanatory value of landscape scale variables in the context of other groups of variables driving ESS supply (see Table 1). Landscape composition was defined as the quantity, and landscape configuration was defined as the relevant shape or form of different land use/land cover classes [89,102]. Landscape naturalness was defined as the degree of human influence or impact on a natural system [103]. In addition to the selected explanatory variables used by others for landscape naturalness, we rated the land use intensity partly based on Brockerhoff et al. [34] as follows: urban (5), agricultural land (4), plantation forestry (3), open water (2) and primary or secondary natural vegetation (1). We calculated landscape metrics using Fragstats 4.1 [104] and R [99] based on the land use/land cover classes indicated in the following formula and the data source in Table 1. We modified the urbanity indicator from Wrbka et al. [103]:

$$Urbanity=log_{10}\left(\frac{U+A+P+1}{F+SG+W+B+1}\right)$$

where *U* is urban, *A* is agricultural land, *P* is plantation forestry, *F* is forestry, *SG* is shrub and grassland, *W* is open water, and *B* is barren land.

**Table 1:** Potential variables explaining ESS supply.

Independent variable	Unit	Methodological reference (data source)
*Landscape composition*		*([61,62])*
Shannon’s diversity of land use/land cover	[*score*]	[128]
Largest patch index	[%]	[104,105,129]
Edge density	[m ha^-1^]	[102,104,125,129]
Share of land use types in the neighborhood:		
Forest	[%]	[14,96]
Agricultural land	[%]	[14,96]
Pine plantation share *(only Satilla watershed)*	[%]	
Corn and winter wheat *(only Big Sunflower watershed)*	[%]	
Wetlands	[%]	[14,96]
*Landscape configuration*		*([61,62])*
Connectance index	[%]	[104]
Effective mesh size	[ha]	[102–104,125]
Landscape shape index	[*score*]	[103,104,125,129]
Distance to stream	[m]	[14]
*Topography*		
Elevation	[m]	[103] ([63])
Slope	[%]	[14,96,103] ([130])
Curvature	[*score*]	[103] ([63])
Aspect	[°]	([131])
*Soil parameters*		[14] ([65])
Saturated hydraulic conductivity	[μm s^-1^]	
Depth to water table	[mm]	
Available water holding capacity	[cm cm^-1^]	
Silt content	[%]	
Soil erodibility	[Mg ha MJ^-1^ mm^-1^]	
*Naturalness*		
Land use intensity	[*score*]	Based on Brockerhoff et al. [34] ([61,62])
Urbanity	[*score*]	Modified from Wrbka et al. [103] ([61,62])
Hemeroby index (human impact)	[*score*]	[125] ([132])

We applied a redundancy analysis (RDA) to identify explanatory values of plot and landscape factors on the variability of ESS supply in the selected case study regions. We chose the RDA because it allows us to (i) estimate the impact of the explanatory variable on ESS supply and vertebrate species richness simultaneously. A more complex alternative, machine learning methods, e.g., boosted regression trees, may only be applied to one response variable [98]. (ii) RDA allows to control for multicollinearity among explanatory variables [95,105]. To consider nonlinear relationships between explanatory variables, we tested also the second degree terms of the potential explanatory variables as recommended by Borcard et al. [106]. We reduced the number of explanatory variables based on the permutation of p-values (p<0.05; 1000 permutations per step) as described by Blanchet et al. [107], which is used instead of the Akaike information criterion (AIC) to select explanatory variables. We used the former method as it delimits the type I error and provides reliable results for non-orthogonal and non-independent explanatory variables [107]. We partitioned the variation into the following groups: plot indicators (topography and soil properties), indicators on landscape composition, landscape configuration and naturalness. To test for spatial autocorrelation, we added the latitudinal and longitudinal coordinates and their interaction as an additional group [95].

### Sites of sufficient and insufficient ecosystem service supply

Villa et al. [108] argued that the benefits from ESS to society are particularly relevant if thresholds or target values, e.g., regarding drinking water quality or good ecological status, are closely met or exceeded. Therefore, we use these thresholds, if available, to distinguish sites of sufficient and insufficient ESS supply. If thresholds are set following representative stakeholder consultation, it may be assumed that they reflect the demand for regulating ESS. In the context of bioenergy, it has been shown that common tools for assessing environmental sustainability, i.e., certification schemes, largely miss such thresholds [12].

The thresholds must be identified at the impact scale of the beneficiaries of ESS, which is the global scale for carbon storage as a factor influencing the global climate. By contrast, the regional or watershed level is relevant for P and sediment retention and groundwater recharge [108]. Typically, P and sediment loading thresholds are set as the *Total Maximum Daily Loadings* for most of the surface water pollutant and are translated to land-based thresholds, e.g., sediment yields/soil erosion rates and P export rates, as indicated in Table 2. Because sediment loadings do not have thresholds in the Satilla watershed as a minor environmental concern, we did not include sediment export as an indicator for identifying sites of sufficient and insufficient ESS supply in the Satilla watershed.

**Table 2:** Sustainability thresholds for P and sediment export set by environmental protection agencies in Georgia and Mississippi with public consultation.

Thresholds	Value	Unit	Data source
P export (Satilla Watershed)	917,627	[lbs a^-1^]	[133]
	2.31	[kg ha^-1^ a^-1^]	
P export (Big Sunflower watershed)	17,759.7	[lbs d^-1^]	[134]
	7.56	[kg ha^-1^ a^-1^]	
Sediment yield (Big Sunflower watershed)	0.6–1.6	[t km^-^² d^-1^]	[135]
	2.19	[Mg ha^-1^ a^-1^]	

Carbon storage of the current land use/land cover was compared with potential natural vegetation [35], following West et al. [109], i.e., sites of sufficient ESS supply are those with a gain in carbon storage for the current land use/land cover toward potential natural vegetation. Such a conservative classification of sites of sufficient and insufficient carbon storage should avoid that the conversion of naturally high carbon stocked land cover types, such as native forests, is viewed as beneficial.

In contrast to the investigated regulating ESS, P and sediment retention, groundwater recharge is an ESS that requires longer time scales to be generated. Therefore, land use activities may have longer lag phases before the consequences become apparent. Groundwater resources are declining due to human groundwater abstraction at both study sites [110–112]. Therefore, higher recharge rates are beneficial. Biodiversity may support other ESS or may be an ESS itself, as discussed in the materials and methods section. Biodiversity as a cultural ESS is highly subjective and strongly varies between stakeholder groups, i.e., among farmers, nature conservation activists, other citizens [113], species or species groups [114]. Facing these limitations, we use the arithmetic mean for both groundwater recharge and biodiversity as the indicative threshold between sufficient and insufficient supply, i.e., assuming that a higher supply is more beneficial.

To explain differences between sites of sufficient and insufficient ESS supply as defined in the materials and methods section, we followed Qiu and Turner [14] and set beneficial sites to one and non-beneficial sites to zero and applied a binomial logistic regression model. We conducted a binomial logistic regression in addition to the RDA since it (i) reflects the case of ESS supply and biodiversity supply relevant in practice, i.e., above and below a threshold or target value and (ii) allows to identify the direction of the impact, i.e., positive or negative on ESS supply. (iii) It is computationally more feasible for larger datasets as in this study than machine learning techniques, e.g., boosted regression trees, and (iv) commonly used in ESS research [98]. We focused on maximizing the bundle of relevant ESS instead of a single ESS. Maximizing bundles of ESS, particularly if regulating services are included, is more likely to ensure the stability of ESS supply, e.g., during sudden changes in environmental conditions. Maximized bundles may also avoid strong tradeoffs toward maximizing single ESS [115]. We removed non-significant explanatory (p<0.05) variables in a backward stepwise manner based on the AIC. Next, variables with variance inflation factors >10 were removed to reduce multicollinearity. The significance of the final model was tested against a null model using a likelihood ratio test. We used the same indicators as those for the RDA to differentiate between sites of sufficient and insufficient ESS supply.

## Results

### Ecosystem service supply in the Satilla and Big Sunflower watersheds

**Fig 2:**
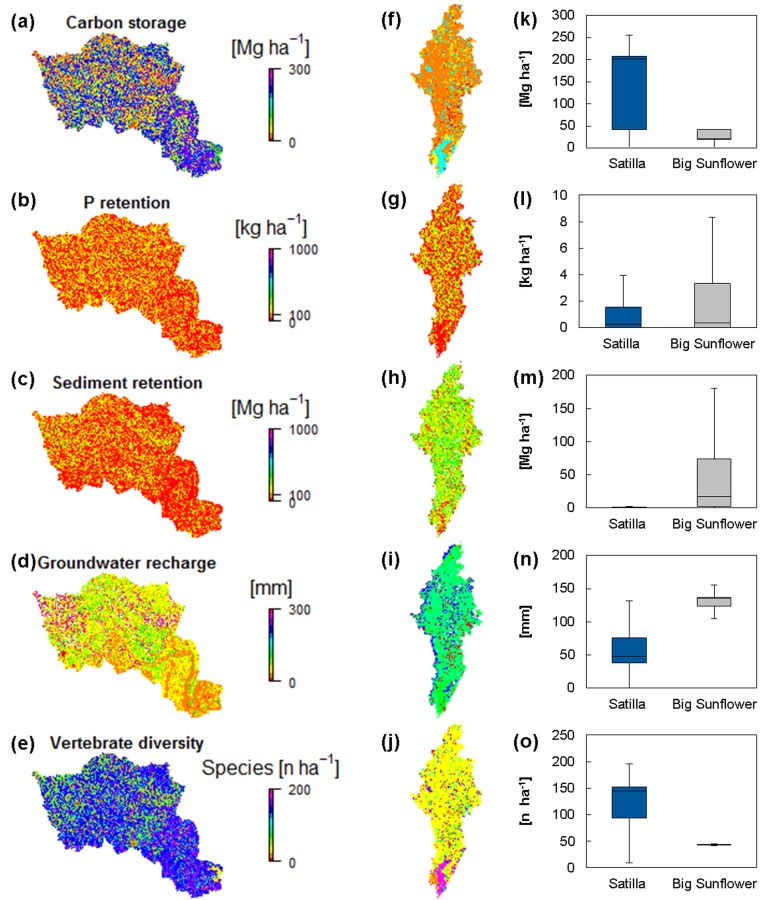
Mapped ESS supply in the Satilla (a-e) and Big Sunflower (f-j) watersheds, also shown as boxplots (k-o). Mapped P and Sediment retention are plotted with breaks at 0.1, 1, 10 and 100 for better visualization.

Examining the plantation forestry system (Satilla watershed) *(left)* and the agricultural production system (Big Sunflower watershed) *(right)*; c.f. Fig. 2A, E, F, J, K, and O., we observed that carbon storage and vertebrate diversity were much higher in the Satilla watershed. In contrast, groundwater recharge and sediment retention were mostly higher in the Big Sunflower watershed; c.f. Fig. 2C, D, H, I, M, and N. P retention was only slightly higher but varied more in the Big Sunflower watershed. If burning was avoided, the potential GHG emission would be reduced by up to 34,000 t for winter wheat and 130,000 t for corn (CO_2_-equivalents (2006); see **Table 3**.

**Table 3:** Potential sustainable biomass availability (calculations based on [38,39,49,50,136,137]) and emission savings in t CO_2_ equivalent (emissions factors (CO_2_ and CH_4_, [51]) for the Mississippi Delta in 2006.

	Sustainable residue removal rates	Potentially available residues				Potential GHG emission savings (residue burning)	
		*lower estimate*		*upper estimate*		*lower estimate*	*upper estimate*
	[%]	DM [t]	HHV [GJ]	DM [t]	HHV [GJ]	CO_2_ eq. [t]	CO_2_ eq. [t]
Winter wheat	15–50	5,900	110,000	20,000	400,000	34,000	20,000
Corn	40–50	90,000	2,000,000	100,000	2,000,000	130,000	110,000

Wheat and corn residues in the Mississippi Delta may contribute up to 0.4% of the potentially available residues of 27 million t dry matter in the entire US in 2012 [39].

**Fig 3:**
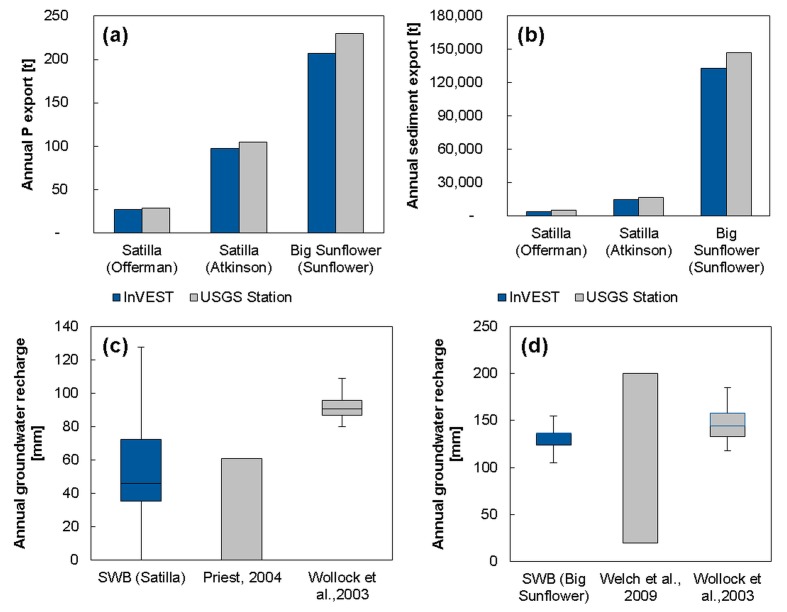
Validation of modeled annual P (a) and sediment export (b) with measured water quality parameters, total P and total suspended solids, and annual groundwater recharge rates with existing studies (c-d). A Turkey boxplot is used for the groundwater recharge rates from an existing model [90] and a range is indicated for existing studies [91,92]. The station and watershed names are listed in brackets.

The modeled annual P export rates explained approximately 90% of the average P concentrations from the empirical data (Fig. 3A). The modeled sediment export rates explained between 78 and 90% of the total suspended solid concentration (Fig. 3B). The modeled groundwater recharge rates for both watersheds were in the range of the existing studies, as shown in Fig. 3C, D.

### Tradeoffs of ecosystem service supply

**Fig 4:**
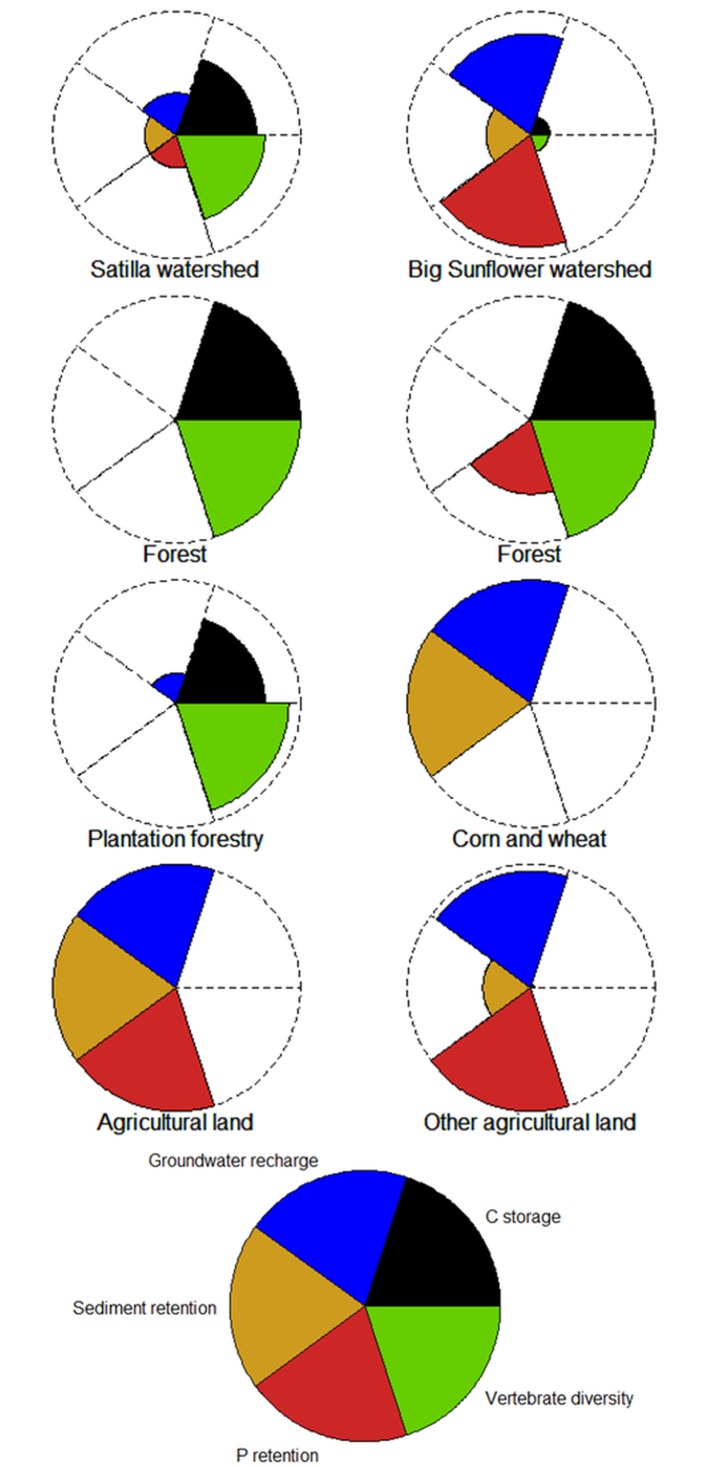
ESS supply (arithmetic mean) for the entire watershed (a, e), natural or semi-natural forest as counterfactual (b, f) for plantation forestry (c) and corn and wheat production (g). The highest arithmetic mean value for each ESS category is used maximum to scale the radar charts for the Satilla (a-d) and Big Sunflower watersheds (e-h) separately.

In the Satilla watershed, plantation forestry had a slightly lower mean carbon storage and vertebrate diversity than did natural- or semi-natural forests and the counterfactual; c.f. Fig. 4B, C. The plantation forestry had a higher vertebrate diversity and carbon storage than did the agricultural land and watershed average; c.f. Fig. 4A, D. By contrast, groundwater recharge was higher for plantation forestry than for forests. The P and sediment retention were negligible compared with agricultural land and the watershed average for both plantation forestry and forests. A paired correlation analysis for a sample of 10,000 pixels from all land use/land cover classes showed a high positive correlation between carbon storage and vertebrate diversity (S1 Fig.). A high negative correlation between groundwater recharge and both vertebrate diversity and carbon storage can be observed.

In the Big Sunflower watershed, corn and wheat production had a significantly lower mean carbon storage, vertebrate diversity and P retention than did forests (Fig. 4F, G). By contrast, the sediment retention and groundwater recharge were higher for corn and wheat production than for forests. A paired correlation analysis for a sample of 10,000 pixels from all land use/land cover classes showed a high positive correlation between carbon storage and vertebrate diversity (S2 Fig.). A lower negative correlation between groundwater recharge and both vertebrate diversity and carbon storage can be observed.

### The influence of topography and soil properties, landscape composition, configuration and naturalness on ecosystem service supply

**Fig 5:**
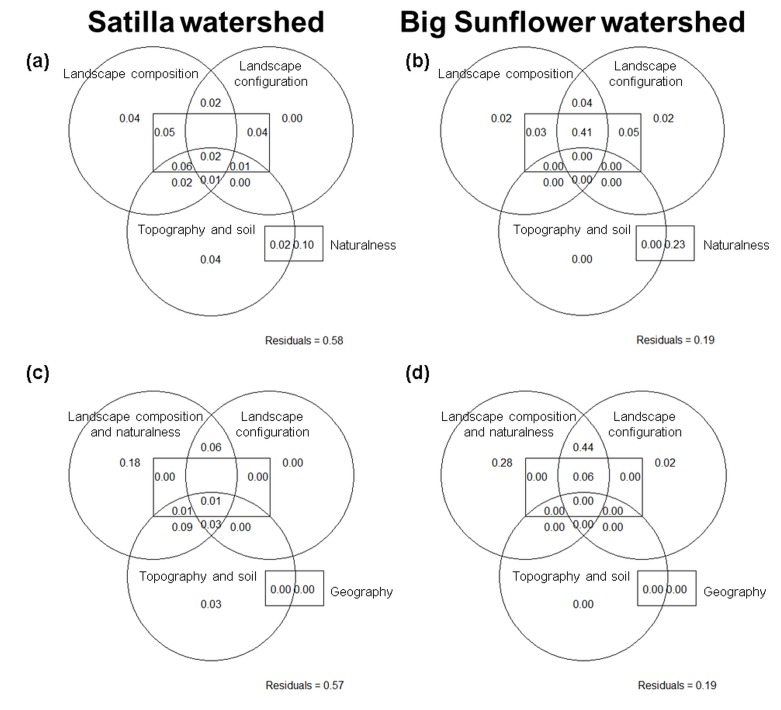
Variation partitioning for ESS supply in the Satilla (a, c) and Big Sunflower (b, d) watersheds without geographic location (a-b) and with geographic location (c-d). p<0.01 and values <0 are not shown; the indicated values display the variance captured by the indicator groups on landscape composition, naturalness, landscape configuration, topography and soil parameters selected from Table 1; the indicators were selected based on permutation p-values; the variance is captured as adjusted R² for single (non-overlapping areas) and combined categories (overlapping areas).

The explained variation in ESS supply for both watersheds did not differ if the combined latitudinal and longitudinal information was included, c.f. Fig. 5C, D, or excluded, c.f. Fig. 5A, B.

The RDA without geographic information for the Satilla watershed (Fig. 5A) as a plantation forestry system showed that the landscape naturalness, i.e., the land use gradient and the hemeroby index (human impact), only explained 10%, and if combined with other indicator groups, a further 20% of the variation in the ESS supply was explained. Landscape composition only explained 4%, and if combined with other indicator groups, 18% of variation in the ESS supply was explained. The selected indicators are the largest patch index for agricultural land and shrub- and grassland. The landscape configuration explained less than 1%, and if combined with other indicator groups, 10% of the variation was explained. The selected indicators are the effective mesh size of shrub- and grassland and water bodies as well as the landscape shape index of forest, plantation forestry and agricultural land. The topography and soil factors explained 4%, and if combined with other indicator groups, 14%.

The RDA without geographic information (Fig. 5B) for the Big Sunflower watershed as an agricultural system showed that the landscape naturalness, i.e., the land use gradient, explained 23%, and if combined with other groups, a further 49% of the variation in the ESS supply was explained. The landscape composition explained 2%, and if combined with other groups, explained a further 48%. The selected indicators are the largest patch index for agricultural land, urban land and water, the edge density for forest and for other land use/land cover categories as well as the Shannon’s diversity of land use/land cover. The landscape configuration and other indicator groups explained 52% of the variation. The selected indicators are the effective mesh size for forest and the landscape shape index for agricultural land. Topography and soil factors were of minor importance.

### Plot and landscape characteristics to distinguish sites of sufficient and insufficient ESS supply

In total, 0.2% of the area of the Big Sunflower watershed had sufficient ESS supply, and 0.9% had insufficient ESS supply. The results for the overall area are shown in the previous section. Corn and wheat production, i.e., 4% of the area of the Big Sunflower watershed, accounted for 1% of the sites of sufficient ESS supply and 5% of the sites of insufficient ESS supply. The major landscape scale factors that promoted sufficient ESS supply were a higher effective mesh size of forests, a higher landscape shape index for agricultural land and a higher edge density of shrub, grassland and water bodies (Table 4). By contrast, a higher land use intensity, a higher edge density of forests and higher landscape shape indices for urban land promoted insufficient ESS supply at the landscape scale. A higher share of corn and wheat production slightly favored insufficient ESS supply. At the plot scale, the higher slope may be associated with insufficient ESS supply.

**Table 4:** Factors characterizing sufficient and insufficient ESS supply in the Big Sunflower watershed (backward logistic regression).

Explanatory variable	Stand. β	SE	z value	Pr(>|z|)	
(Intercept)	0.2938	0.4829	0.608	0.54288	
***Topography and soil parameters***					
Elevation [m]	-6.6488	0.2702	-24.605	< 0.0001	***
Slope [%]	-12.0045	0.3313	-36.235	< 0.0001	***
Silt content [%]	2.5642	0.2503	10.244	< 0.0001	***
Saturated hydraulic conductivity [μm s^-1^]	2.5663	0.5208	4.928	< 0.0001	***
Soil erodibility	-0.6872	0.1632	-4.212	< 0.0001	***
Depth to water table [mm]	3.4742	0.2796	12.426	< 0.0001	***
Available water holding capacity [cm cm^-1^]	-0.8073	0.2871	-2.812	0.00493	**
***Naturalness***					
Land use intensity	-6.0203	0.4517	-13.329	< 0.0001	***
Hemeroby	-0.975	0.2592	-3.761	0.00017	***
***Landscape composition***					
Corn/Wheat production, 300 m buffer [%]	-0.8912	0.3559	-2.504	0.01228	*
Edge density (forest)	-1.7479	0.3269	-5.347	< 0.0001	***
Edge density (shrub and grassland)	10.4326	0.8355	12.487	< 0.0001	***
Edge density (open water)	5.0185	0.6683	7.509	< 0.0001	***
Largest patch index (urban)	1.3618	0.3367	4.045	< 0.0001	***
***Landscape configuration***					
Effective mesh size (forest)	10.904	1.2152	8.973	< 0.0001	***
Landscape shape index (agricultural land)	11.4842	0.6129	18.737	< 0.0001	***
Landscape shape index (open water)	3.9223	0.3983	9.848	< 0.0001	***
Landscape shape index (urban)	-2.6192	0.2023	-12.947	< 0.0001	***

A positive value for the standardized β indicates that an explanatory variable is contributing to sufficient ESS supply; a negative value for the standardized β indicates that an explanatory variable is contributing to insufficient ESS supply.

In total, 0.1% of the area of the Satilla watershed showed sufficient ESS supply, and 0.6% showed insufficient ESS supply. The plantation forestry, i.e., 28% of the area of the Satilla watershed in 2006, accounted for 19% of the sites of sufficient ESS supply and for 1.6% of the sites of insufficient ESS supply. The major landscape scale factors that were favorable for a sufficient ESS supply were a higher edge density as well as a high largest patch index of forest and plantation forestry (S1 Table). At the plot scale, a higher available water holding capacity and a higher depth to water table were beneficial. In contrast, a higher landscape shape index of forests, a greater land use intensity, a higher share of wetlands and a higher largest patch index of agricultural land promoted insufficient ESS supply at the landscape scale.

The likelihood ratio tests showed a significant difference when compared to a null model for the Big Sunflower watershed (χ^2^ = 20440, df = 18, p<2.2e-16) and the Satilla (χ^2^ = 35944, df = 22, p<2.2e-16) (the set of explanatory variables shown in Table 4 and S1 Table had a significant explanatory value when compared against the model without explanatory variables).

## Discussion

### Ecosystem service supply synergies and tradeoffs

The higher carbon storage and biodiversity in the Satilla watershed may be related to the higher resemblance of plantation forestry to natural or semi-natural forest, see [34]. Higher P and sediment retention in the Big Sunflower watershed are attributable to the higher P application and bare soil in agricultural production systems, resulting in a higher demand for related regulating services, see Fig. 3A, B. The higher rate of groundwater decline in the Big Sunflower watershed [112] hints at a stronger mismatch of groundwater abstraction and recharge compared with the Satilla watershed.

Generally, tradeoffs for agricultural production systems have been shown in other studies, e.g., [14,15,116,117], but not for plantation forestry or for the intensive feedstock production systems evaluated in this study. Overall, the plantation forestry deviates less from the counterfactual natural or semi-natural forest than from agricultural production systems such as corn and wheat; thus, we conclude that the plantation forestry system is preferable for the modeled ESS bundle and biodiversity.

### Advantages and constraints of the ecosystem service modeling scheme

We achieved the models’ purpose of providing a broad picture of water quality [118] and of assessing annual groundwater recharge rates [88]. For such application, we achieved a reasonably good validity (Fig. 3); our results are comparable with those of Qiu and Turner [14] and Terrado et al. [119]. In addition, simple tools such as InVEST are more likely to be an option for practitioners for regional scale assessments [118], e.g., for bioenergy, agricultural or forestry production systems, compared to significantly more complex and resource-intensive models such as the Soil-Water Assessment Tool (SWAT) [120]. For example, InVEST may be used in data scarce situations without monthly or daily precipitation data, the latter required by SWAT for nutrient and sediment modeling. Therefore, this study design is suitable for regions with high or low data availability, which will facilitate comparable analyses for bioenergy production systems around the world to compare ESS supply and biodiversity, e.g., within this study. However, tools such as InVEST or SWB do not support simulation studies requiring numerous runs to find Pareto optimal solutions of ESS supply, e.g., [121], or biodiversity, e.g., [122].

Considering only the higher biomass yield of pine plantations and the lower yields from corn stover and cereal straw [39], the smaller tradeoffs of pine plantations may be seen as preferable than agricultural biomass at first glance. Bennett and Balvanera [16] similarly argued that the tradeoffs of provisioning ESS should be minimized to ensure a balanced ESS supply. Focusing on the yield, i.e., for bioenergy, and the environmental side may disregard the social side of the “food, energy and environment trilemma” [21]. This trilemma may be solved if (i) there are no competing uses of wheat straw or corn stover [21,39] and (ii) if both corn stover and wheat straw are produced regardless; the identified ESS tradeoffs with forests do not exclusively need to be attributed to a potentially used residual biomass.

### Benefits of avoided and potential further competition for biomass

Due to the missing competing uses for the agricultural residues in the Mississippi Delta, their potential use for bioenergy instead of burning may create synergies. For example, (i) negative impacts on soil structure, local microbiology, the water holding capacity of soil and soil fertility from burning could be avoided [123,124], and (ii) we could reduce GHG emissions or at least generate energy as an additional use. However, the actual sustainable residue removal rates and GHG emissions depend on the local environmental conditions, e.g., soil organic carbon or water availability and conversion and use aspects, e.g., the harvesting technique, transportation and the type of energy carrier. Therefore, further studies may integrate results from this study into life-cycle assessments to compare these iLUC free bioenergy feedstocks with a fossil fuel energy carrier. For the plantation forestry system, future research may investigate (i) the global production pattern changes due to the decline of the pulp and paper industry in the southeastern US to identify potential indirect impacts, such as iLUC, and (ii) the ESS supply and tradeoffs of current and future pulp and paper exporting regions of the world that partly substitute production capacities in the southeastern US and that may be of interest; e.g., Eucalyptus plantations in Brazil or some parts of sub-Saharan Africa may reveal other economic, social or environmental issues.

### Factors explaining the variance of ecosystem service supply

We confirmed the hypothesis of Frank et al. [125] that the degree of naturalness of the landscape affects ESS supply. Wrbka et al. [103] previously demonstrated this relation for the human appropriation of net primary productivity, e.g., biomass use. The impact of landscape composition and configuration on ESS supply was only slightly less important, as proposed by Syrbe and Walz [102] and Frank et al. [125], e.g., the largest patch index, the effective mesh size or the landscape shape index in both watersheds. For example, in the Satilla watershed, the substantial influence of the largest patch index of agricultural land and of the effective mesh size of shrub and grassland habitats have shown that not only the existence but also the location and connection of shrub and grassland patches are important for ESS supply. Further influential factors related to the climate are not considered in this study because both case studies are in the same humid subtropical climate zone according to Koppen-Geiger [126]; however, this may be reasonable for case studies with climatic gradients, e.g., [95], or when comparing case studies in different climate zones, e.g., a humid subtropical climate compared with a semi-arid tropical climate.

### Thresholds and local demand for ecosystem services

To link ESS supply to the local demand, we related the actual supply to locally set environmental thresholds when available for sediment and P concentrations in surface waters, as in Terrado et al. [119]. Considering local preferences may more likely allow us to determine whether ESS provide a human benefit. However, this consideration is not regularly performed in ESS modeling, see, e.g., [3,15]. Such socially accepted environmental thresholds for groundwater recharge or biodiversity.

When comparing the sites above and below thresholds, the plantation forestry contributed to sufficient ESS supply. In the Big Sunflower watershed, ESS supply may be enhanced by a higher complexity of agricultural patches. It simultaneously requires combining agricultural land with, e.g., shrub and grassland or forest patches rather than urban land, as shown by the negative impact of increasing land use intensity. This is supported by the beneficial effect of an increase in size and number of shrub and grassland patches. It is indirectly shown by the beneficial effect of a higher edge density and a higher effective mesh size of forest patches. In the Satilla watershed, it may be beneficial to increase the size and connectivity of forest patches. By contrast, it does not seem beneficial to enhance the complexity of forest patches. This may be explained by the fact that more complex forest patches have a larger share of non-forest land use, e.g., pine plantations, agricultural or urban land. These results are in line with the results of the tradeoff analysis showing the higher supply of carbon storage and biodiversity toward plantation forestry. However, a higher dominance of agricultural land decreases ESS supply, whereas plantation forestry still increases ESS supply. Future research should assess the relevance of landscape composition and configuration and naturalness of the landscape in other solid biomass production systems in other parts of the world.

In practice, the rules of certification schemes or the rules set by local authorities in landscape planning should include rules on landscape composition and configuration. It may be reasonable to consider an additional assessment of ESS supply at the landscape scale in improved certification schemes, e.g., for bioenergy. An assessment at the plot scale of the individual feedstock producer seems incomplete. Even if the assessment would go beyond the management practices, the local environmental factors, topography and soil parameters explained only a small share of the variation in ESS supply. For example, preserving or creating a landscape mosaic may better balance the supply of ESS beyond the bioenergy feedstock in the production region. One concrete option could be a higher number of connected forest patches as buffer strips alongside rivers in the Big Sunflower watershed or other natural vegetation, such as grassland in agricultural watersheds [31]. For the Satilla river, we may see forested buffer strips in Fig. 1, which are required in Georgia’s Mountain and River Corridor Protection Act [127]. Another strategy to reduce the intensity of agricultural production could be 2^nd^ generation bioenergy feedstocks, such as perennial bioenergy grasses or short rotation coppice species. Such bioenergy-providing species may provide both biomass and higher ESS (sediment and nutrient retention) and biodiversity, c.f. [4,31].

### Potential future use of indicators on landscape structure

We suggest including indicators of landscape structure to ensure a harmonized level of sustainability in certification schemes, which is particularly relevant if the biomass is largely traded, e.g., wood pellets in the form of pine plantations in the southeast US; they should also be subjected to legal frameworks, which are currently absent or variable, for landscape planning. For a broader application in sustainability assessments beyond bioenergy, the impact of landscape structure on ESS supply should be tested in other agricultural or forest production systems.

## Supporting Information

S1 Fig Paired correlation analysis of ESS supply in the Satilla watershed (p<0.001 (***); p<0.01 (**), p<0.05 (*)). (TIF)

S2 Fig Paired correlation analysis of ESS supply in the Big Sunflower watershed (p<0.001 (***); p<0.01 (**), p<0.05 (*)). (TIF)

S1 Table Factors characterizing sufficient and insufficient ESS supply in the Satilla watershed (backward logistic regression). A positive value for the standardized β indicates that an explanatory variable is contributing to sufficient ESS supply; a negative value for the standardized β indicates that an explanatory variable is contributing to insufficient ESS supply.
(TIF)
